# Magnetic Nanoparticle‐Assisted Non‐Viral CRISPR‐Cas9 for Enhanced Genome Editing to Treat Rett Syndrome

**DOI:** 10.1002/advs.202306432

**Published:** 2024-04-22

**Authors:** Hyeon‐Yeol Cho, Myungsik Yoo, Thanapat Pongkulapa, Hudifah Rabie, Alysson R. Muotri, Perry T. Yin, Jeong‐Woo Choi, Ki‐Bum Lee

**Affiliations:** ^1^ Department of Chemistry and Chemical Biology Rutgers, The State University of New Jersey Piscataway NJ 08854 USA; ^2^ Department of Chemical and Biomolecular Engineering Sogang University Seoul 04107 South Korea; ^3^ Department of Bio and Fermentation Convergence Technology Kookmin University Seoul 02707 South Korea; ^4^ W. M. Keck Center for Collaborative Neuroscience and Department of Cell Biology and Neuroscience Rutgers, The State University of New Jersey Piscataway NJ 08854 USA; ^5^ School of Medicine Department of Pediatrics/Rady Children's Hospital San Diego Department of Cellular and Molecular Medicine Stem Cell Program La Jolla CA 92093 USA; ^6^ Department of Biomedical Engineering Rutgers The State University of New Jersey Piscataway NJ 08854 USA

**Keywords:** CRISPR‐Cas9, genome editing, magnetic nanoparticle, non‐viral, Rett syndrome

## Abstract

The CRISPR‐Cas9 technology has the potential to revolutionize the treatment of various diseases, including Rett syndrome, by enabling the correction of genes or mutations in human patient cells. However, several challenges need to be addressed before its widespread clinical application. These challenges include the low delivery efficiencies to target cells, the actual efficiency of the genome‐editing process, and the precision with which the CRISPR‐Cas system operates. Herein, the study presents a Magnetic Nanoparticle‐Assisted Genome Editing (**MAGE**) platform, which significantly improves the transfection efficiency, biocompatibility, and genome‐editing accuracy of CRISPR‐Cas9 technology. To demonstrate the feasibility of the developed technology, MAGE is applied to correct the mutated MeCP2 gene in induced pluripotent stem cell‐derived neural progenitor cells (iPSC‐NPCs) from a Rett syndrome patient. By combining magnetofection and magnetic‐activated cell sorting, MAGE achieves higher multi‐plasmid delivery (99.3%) and repairing efficiencies (42.95%) with significantly shorter incubation times than conventional transfection agents without size limitations on plasmids. The repaired iPSC‐NPCs showed similar characteristics as wild‐type neurons when they differentiated into neurons, further validating MAGE and its potential for future clinical applications. In short, the developed nanobio‐combined CRISPR‐Cas9 technology offers the potential for various clinical applications, particularly in stem cell therapies targeting different genetic diseases.

## Graphical abstract

This study presents a novel Magnetic Nanoparticle‐Assisted Genome Editing (MAGE) platform that improves the delivery and genome editing capabilities of CRISPR‐Cas9 for treating Rett syndrome. This method involves using magnetic nanoparticles to sort patient‐derived cells after non‐viral plasmid delivery, significantly enhancing gene‐repairing efficiency and biocompatibility. The MAGE platform shows promise for stem cell therapies and the treatment of genetic disorders.

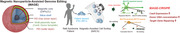

## 1 Introduction

The increase in the prevalence of genetic disorders has driven the necessity of developing advanced therapeutic strategies, including genome editing therapy. Currently, over 7000 known genetic disorders affect ≈1 in 21 people worldwide.^[^ ^1^ ^]^ According to the U.S. Food and Drug Administration, only ≈5–7% of rare diseases have an FDA‐approved therapy for their treatment.^[^ ^2^ ^]^ These findings underscore a rising demand for complex biological therapies (e.g., personalized medicine) for these disorders. The emergence of clustered regularly interspaced short palindromic repeats (CRISPR)/Cas9‐based genome editing and induced pluripotent stem cell technologies have shifted the paradigm of biological drug development trend toward an ex vivo genome editing approach using patient‐derived stem cells.^[^ ^3^ ^]^ Compared to in vivo methods, this approach is believed to offer superior advantages to minimize off‐target gene editing. Additionally, it provides an opportunity to screen for successful gene modifications. These features collectively contribute to the enhanced safety benefits of this method.^[^ ^3^, ^4^ ^]^

Most CRISPR delivery platforms utilized in clinical, manufacturing, and research settings are based on viral vectors. Historically, recombinant viral vectors have been the leading approach to human gene therapy due to their high efficacy and long‐term expression of transgenes. Among many viral vectors, adeno‐associated viruses (AAVs) and lentiviruses are the most widely used vectors in clinical studies, dominating in vivo and ex vivo gene therapy clinical trials, respectively.^[^ ^5^ ^]^ However, these viral vectors have limited packaging ability and can provoke mutagenesis or carcinogenesis. As such, there has been an increasing interest in developing non‐viral delivery methods as an alternative to viral methods, including using nanocarriers composed of lipids or cationic polymers.^[^ ^6^ ^]^ In contrast to traditional viral methods, non‐viral methods offer several distinct benefits. These include the ability to transport large‐sized genetic materials, reduced potential for triggering immune responses, and eliminating risks associated with endogenous virus recombination. In addition, non‐viral methods have been investigated to increase the efficiency of CRISPR delivery by utilizing the inherent capabilities of non‐viral delivery vehicles, such as magnetofection with magnetic nanoparticles.^[^ ^7^ ^]^ Collectively, these features decrease the likelihood of both short‐term and long‐term adverse reactions. However, despite these advantages, non‐viral delivery methods are plagued by low editing efficiencies that must be overcome before being translated to the clinic, especially in stem cell therapy for genetic diseases.^[^ ^8^ ^]^

**Figure 1:**
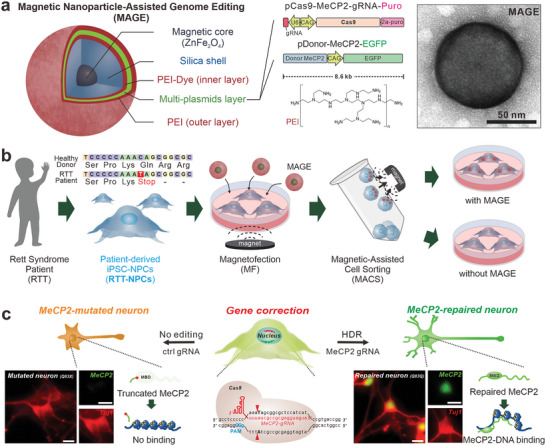
A magnetic nanoparticle‐assisted gene editing (MAGE) platform for repairing Rett syndrome in patient‐derived stem cells with non‐viral CRISPR‐Cas9 delivery. a) The MAGE comprises a magnetic nanoparticle, two plasmids of CRISPR‐Cas9 and donor DNA for gene editing, and cationic polymers. b) MAGEs are treated to genetic disorder (Rett syndrome, RTT) patient‐derived induced‐neural progenitor cells (iPSC‐NPCs_(Q83X)_) and sorted by magnetic‐activated cell sorting (MACS) to collect the iPSC‐NPCs_(Q83X)_ that possess the plasmids. c) MAGE‐contained cells are repaired via homology direct repair and express the MeCP2 protein while patient cells do not. Red: Tuj1, Green: MeCP2. The scale bar is 40 µm.

Addressing this, we have developed a novel magnetic nanoparticle‐assisted genome editing (MAGE) platform, using a magnetic core‐shell nanoparticle (MCNP) to deliver multiple plasmids encoding the CRISPR‐Cas9 system with a magnetic force (**Figure** **1**a,b). The underlying concept is that magnetofection first enables improved multi‐plasmid cell delivery, and plasmids‐containing cells can then be purified by magnetic‐activated cell sorting (MACS) (Figure 1b). This results in an increased expression of both the Cas9 protein and gRNA, leading to a successful modification of the target mutant gene. This modification is achieved through homology‐directed gene repair using the accurate donor DNA sequence. This innovative platform offers several advantages, such as i) faster and more efficient delivery of multiple genes due to magnetofection, ii) MACS, iii) enhanced biocompatibility, and iv) in situ, real‐time tracking of the delivery process, which all synergistically enhance the gene repairing efficiency of CRISPR‐Cas9.

Interestingly, such non‐viral methods (magnetofection and MACS) have gained attention. They are individually used to enhance the efficiency of CRISPR delivery by leveraging the inherent properties of non‐viral delivery vehicles. However, our novel method, MAGE platform‐based CRISPR‐Cas9 (MAGE‐CRISPR), offers several distinct advantages over previous magnetofection and MACS‐based techniques. First, our method incorporates a specialized nanoparticle design (magnetic core‐shell nanoparticle with complementary outer shell) that allows for better protection of the CRISPR‐Cas9 components during the delivery process, minimizing degradation and increasing the stability of the system. This increased stability leads to more consistent and reliable genome editing results compared to previous magnetofection approaches. Second, MAGE‐CRISPR offers a more streamlined and user‐friendly workflow compared to conventional MACS‐based methods. By eliminating the need for complex cell sorting procedures, our method reduces the time and resources required for genome editing, making it more accessible and scalable for potential clinical applications.

Collectively, MAGE‐CRISPR represents a significant advancement over previous magnetofection and MACS‐based methods for non‐viral CRISPR delivery. Our method's unique combination of magnetic nanoparticles and non‐viral vectors, specialized nanoparticle design, and streamlined workflow enables enhanced genome editing efficiency, stability, and accessibility, ultimately leading to improved treatment outcomes for genetic disorders.

As a model system, we applied the MAGE‐CRISPR to edit a mutated gene in stem cells derived from a patient with Rett syndrome, a rare genetic disorder caused by *de novo* mutations in the mutated X‐linked methyl CpG‐binding protein 2 (MeCP2) gene (Figure S1, Supporting Information). More specifically, to demonstrate the in vitro patient‐specific treatment of Rett syndrome, we generated induced pluripotent stem cell‐derived neural progenitor cells (iPSC‐NPCs) obtained from a Rett syndrome patient (RTT‐NPCs) and designed two plasmids to cut and replace the mutated MeCP2 gene (Figure 1c). It was then determined that the MAGE‐CRISPR could effectively facilitate the transfection of the CRISPR‐Cas9 plasmid and the isolation of the gene‐edited cells, thereby successfully demonstrating the usefulness of our technology for future clinical applications in autologous stem cell therapy.

## 2 Results and Discussion

### 2.1 Construction of Patient‐Specific CRISPR‐Cas9 Vectors Targeting the Mutated MeCP2 Gene

To design the gRNA and donor sequence for the CRISPR‐Cas9‐based patient‐specific mutated gene repair, we obtained skin fibroblasts from a male Rett syndrome patient whose mutated MeCP2 gene sequence was previously determined and reported.^[^ ^9^ ^]^ In the case of this Rett syndrome patient, the MeCP2 gene has a nonsense mutation in amino acid residue 83, which transforms glutamine (“C”AG) to a premature stop codon (“T”AG), resulting in truncation and degradation of the MeCP2 protein (Q83X) (Figure S1, Supporting Information). To this end, we designed two plasmid systems to edit the mutated MeCP2 genome: a combined single Cas9 gene with gRNA named pCas9‐gRNA‐Puro (pCas9) and a donor sequence with an EGFP reporter gene named pDonor‐MeCP2‐EGFP (pDonor) (Figure S2a,b, Supporting Information). In particular, pCas9 is a single, combined plasmid system designed to express both Cas9 double‐stranded nuclease and a gRNA that was designed to target the mutated site of the MeCP2 gene (MeCP2‐gRNA) as well as a selectable marker puromycin. A gRNA (control‐gRNA) was constructed using an empty sequence of the target gene as a control (Figure S2c,d, Supporting Information). Finally, to prevent the re‐cleavage of repaired MeCP2 by MeCP2‐gRNA and Cas9 after successful editing, a 10‐base pair donor sequence was replaced with a complementary nucleic acid sequence that would not be recognized by the MeCP2‐gRNA and would still translate into the correct MeCP2 amino acid sequence.

### 2.2 Development of the MAGE‐CRISPR Using Functional Nanoparticles

**Figure 2:**
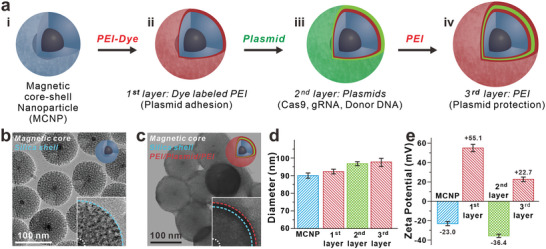
Layer‐by‐layer conjugation of MAGE. a) Schematic diagram of MAGE synthesis process. b‐c, TEM image of the magnetic core‐mesoporous silica shell nanoparticle b) before and c) after the plasmid loading. d) Size of the particle is increased through the synthesis process. e) Each step of synthesis process is confirmed by surface charge change with each layer's unique electrostatic information; silica shell surface (negative), PEI (positive), and plasmids (negative).

To achieve both efficient delivery of CRISPR plasmids to iPSC‐NPCs and monitor cellular uptake, we developed a novel magnetic nanoparticle platform termed MAGE using layer‐by‐layer assembly (**Figure** **2**a). The magnetic properties of the MAGE‐CRISPR are derived from MCNPs with zinc‐doped iron oxide (ZnFe_2_O_4_) cores. This core has previously been shown to have a significantly higher saturation magnetization when compared to conventional Fe_2_O_3_ or Fe_3_O_4_ magnetic nanoparticles (MNPs).^[^ ^10^ ^]^ As such, we first synthesized ZnFe_2_O_4_ cores via the thermal decomposition of a mixture of metal precursors (zinc chloride, ferrous chloride, and ferric acetylacetonate) in the presence of oleic acid and oleylamine using a previously reported protocol that was modified by our group.^[^ ^11^ ^]^ Following core synthesis, an inert silica shell was formed via the condensation of tetraethyl orthosilicate in the presence of a cetrimonium bromide micelle template to improve the colloidal stability and the plasmid loading capacity.^[^ ^12^ ^]^ Transmission electron microscopy (TEM) revealed that the diameter of the cores was 7.93 ± 1.6 nm and that the MNP cores were uniformly coated with a 25.21 ± 3.8 nm thick silica shell (Figure 2b). For more detailed characterization, TEM revealed the monocrystalline structure of the MNP cores with a lattice fringe measured to be 4.8 Å, which is characteristic of the (111) plane of the spinel.^[^ ^11^ ^]^ As a result, the overall diameter of the as‐synthesized MCNPs was 90.12 ± 1.37 nm.

To utilize the aforementioned MCNPs for plasmid delivery, the MCNPs were coated with two layers of branched polyethylenimine (PEI) via electrostatic interactions in the presence of NaCl to afford the MCNPs with an overall positive charge. An initial layer of tetramethylrhodamine (TRITC)‐labeled PEI was coated on the MCNP surface to allow for complexing with plasmid via electrostatic interactions and in situ monitoring of plasmid release from the MCNP surface. After loading the plasmid as a second layer, an additional layer of PEI was applied to protect the plasmid further and improve intracellular delivery/release efficiency. As a result, this would facilitate MCNP complexation with plasmid DNA and induce endosomolysis within the cytoplasm.^[^ ^13^ ^]^ The resulting MAGE‐CRISPR had a final hydrodynamic size of 98.84 ± 3.96 nm (Figure 2c,d) and zeta potential of 22.7 ± 4.66 mV (Figure 2e). To find the optimal concentration of plasmid‐loaded MCNP‐PEI while maintaining cell viability, we tested the cytotoxic effects of different doses of particles and loaded plasmids on patient‐derived RTT‐NPCs (Figure S3, Supporting Information). To minimize cytotoxicity while maximizing transfection efficiency, we used 10 kDa branched PEI, which has previously been demonstrated to be biocompatible with stem cells.^[^ ^14^ ^]^ We found that the MAGE‐CRISPR can load more than 80% of the plasmid with a 1:15 mass ratio (plasmid: MCNP) with minimal cytotoxicity (≤70 µg mL^−1^ MCNP, 96.2% viability). Moreover, mixed populations of the pCas9 and pDonor plasmids can be loaded onto a single nanoparticle, thus allowing co‐delivery of both the CRISPR‐Cas9 system and the donor templates into a single cell using the MAGE‐CRISPR. As such, this platform shows excellent potential for therapeutic genome editing owing to its ability to load multiple plasmids, unlike Adeno‐associated viral particles (AAV).^[^ ^15^ ^]^

### 2.3 Magnetic‐Assisted Multi‐Plasmid CRISPR‐Cas9 Delivery

**Figure 3:**
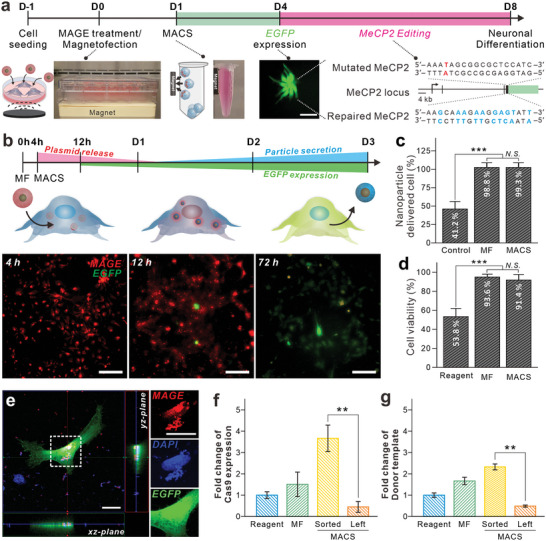
MAGE delivered cell sorting and its gene expression. a) Strategy for MAGE‐mediated gene editing of RTT‐NPCs. White dash‐line circles indicate magnetically captured MAGE‐containing cells. Scale bar: 100 µm. b) Time‐dependent MAGE delivery and delivered plasmid expression. RTT‐NPCs_(Q83X)_ were monitored at different time points (4, 12, and 72 h) with two different fluorescence channels (Red: MAGE, Green: EGFP). c) Quantification of nanoparticle delivered cells population (*t*‐test, ^***^ *P* < 0.001, error bars: s.e.m.). d) Cytotoxicity analysis of MAGE transfected cells (*t*‐test, ^**^ *P* < 0.01, error bars: s.e.m.). e) Confocal microscopic image of RTT‐NPCs_(Q83X)_ 24 h after MAGE transfection. Red: Rhodamine B labeled MAGE, Blue: DAPI, Green: EGFP. Scale bar: 20 µm. f,g) Relative amount of expressed Cas9 mRNA (f) and delivered Donor template (g) in MAGE‐treated RTT‐NPCs_(Q83X)_ compared to the transfection reagent. Reagent, transfection reagent (Lipofectamine 3000); MF, magnetofection; MACS, magnetic‐assisted cell sorting; MAGE, magnetic nanoparticle‐assisted gene editing; EGFP, enhanced green fluorescence protein.

To assess the efficiency of cellular uptake of MAGE, we performed fluorescence microscopy on iPSC‐NPCs derived from the Rett syndrome patient (RTT‐NPCs_(Q83X)_) and healthy donor (wild type, WT‐NPCs) using a previously established method (**Figure** **3**a).^[^ ^9^, ^16^ ^]^ Here, MAGE was assembled using 2 µg of plasmids (total 2 µg, 1 µg each of pCas9 and pDonor), and delivery was enhanced using magnetofection. This well‐established method (magnetofection) allows for the rapid accumulation of MCNPs and their payloads upon exposure to an external magnetic field. From 4 h after transfection, we visualized and quantified the efficiency of uptake with time‐laps imaging (Figure 3b). We found that the RTT‐NPCs_(Q83X)_ could efficiently uptake MAGE with magnetofection (98.8% efficiency) (Figure 3c). Moreover, the MCNPs that remained intracellularly after magnetofection provided cellular mobility in a magnetic field, which allowed for cell sorting via MACS to sort out the cells containing MAGE with 99.3% accuracy. It should be noted that, in contrast to other cationic transfection methods, which can result in low cell viabilities, MAGE, and magnetofection caused little to no cytotoxicity (≈92% cell viability after MAGE delivery and magnetofection) owing to the much lower concentration and shorter time of incubation that is necessary (Figure 3d). Specifically, it was found that most of the MAGE was secreted out from the cell via exocytosis ≈ 48 h after the transfection, which minimized nanoparticle‐mediated cytotoxicity.^[^ ^17^ ^]^

Afterward, compared to commercially available transfection agents, the ratio of cells expressing EGFP due to the magnetofection of pDonor with MAGE was significantly greater (Figure 3e; Figure S4, Supporting Information). The discrepancy between the protein expression results and those presented in Figure 3c is due to our criteria for nanoparticle delivery. Our initial assessment of successful nanoparticle delivery relied solely on the presence of a fluorescent signal, neglecting the quantification of intracellular MAGE content. However, upon assessing the MAGE content in each cell and the copy number of the transferred plasmid, it became apparent that cells with higher quantities of MAGE were selectively sorted using MACS (Figure S5, Supporting Information). These results are closely correlated to the 3.6 folds with higher Cas9 expression level and 2.3 folds more amount of pDonor in RTT‐NPCs_(Q83X)_ after MACS (Figure 3f,g). As respectively, leftover cells were shown no significant level of Cas9 expression. As such, we confirmed that it can efficiently deliver CRISPR‐Cas9 and donor template plasmids to the same cells and sort cells exhibiting a high expression of the transferred genes.

### 2.4 Improving the Efficiency of Mutated MeCP2 Repair Using MAGE

**Figure 4:**
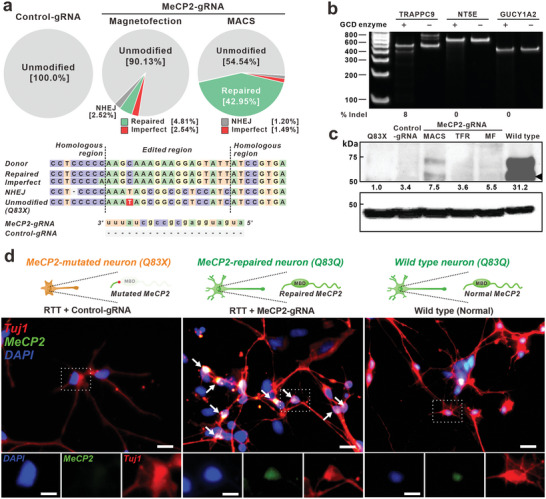
Correction of mutated MeCP2 gene by non‐viral delivered CRISPR‐Cas9. a) Graphical representation of the HDR results with various working conditions. Red box of the unmodified sequence indicates the mutated sequence. b) GCD assay for three representative off‐target candidate genes to confirm the off‐target effect of the MeCP2‐gRNA. c) Western blot of MeCP2 protein expression two weeks after the transfection and quantification of MeCP2 levels in differentiated cells. TFR: Transfection reagent. d) Immunostaining of differentiated neurons after MAGE‐mediated MeCP2 editing. Edited RTT‐neurons express the MeCP2 in the nuclei as same as WT‐neurons while MeCP2 is not shown in control RTT‐neurons. White arrow indicates MeCP2^+^ cells. Scale bar: 20 µm.

The mutated gene can be repaired by delivering donor DNA with CRISPR‐Cas9 through a homology‐directed repair (HDR) pathway.^[^ ^18^ ^]^ Although non‐viral delivery systems for HDR have seen significant advancements, the efficiencies reported in various studies can be inconsistent and differ from one another.^[^ ^19^ ^]^ For example, in the study conducted by Farbiak et al., they reported achieving an efficiency of ≈50% using dendrimer‐based lipid nanoparticles. In contrast, Xu et al. documented an efficiency of 42.1% in induced pluripotent stem cells (iPSCs) by using electroporation,^[^ ^20^ ^]^ and Xu et al. achieved 42.1% in iPSCs using electroporation.^[^ ^21^ ^]^ However, it is pertinent to note that many nanoparticle‐based non‐viral systems for HDR of stem cells have reported efficiencies close to 10% or even single‐digit efficiencies, as in the case of Xie et al. which reported an efficiency of 7%.^[^ ^22^ ^]^ To improve repair efficiency, two different CRISPR‐Cas9 delivery methods with the MAGE‐CRISPR – magnetofection and MAGE – were tested (**Figure** **4**). The verification of MeCP2 genome editing was accomplished by performing molecular analysis at both the DNA and protein levels, including sequencing, western blotting, immunostaining, and downstream gene expression profiling. First, the genome repairing efficiency was calculated based on the next‐generation sequencing results of RTT‐NPCs_(Q83X)_ with MAGE (Figure 4a). Compared with magnetofection, MACS showed a significant increase in HDR efficiency (42.95%) with minimal imperfections (1.49%), while magnetofection alone showed 4.81% HDR efficiency as measured using next‐generation sequencing analysis. The improvement in HDR efficiency resulted from the increase in intracellularly released pDonor, owing to the sorting for MAGE‐containing cells (Figure S5, Supporting Information).

On the other hand, the high content of the Cas9 plasmid in the cell can induce a higher off‐target effect due to the overexpression of Cas9. Using Cas‐OFFinder, an off‐target prediction website, 3 potential off‐target sites were identified with a parameter tolerant to a 3‐bp mismatch. However, as shown in Figure 4b, the gene cleavage detection (GCD) assay showed no significant editing occurred in these sites after HDR with MAGE.

Next, we used a western blot to compare MeCP2 expression levels among plasmid‐treated neurons differentiated from different NPCs (WT, RTT‐_(Q83X)_, edited RTT_(Q83X)_ with control‐gRNA and MeCP2‐gRNA) (Figure 4c). To confirm the expression of full‐length MeCP2, N‐terminus targeting anti‐MeCP2 antibody, which cannot attach to the truncated protein (MeCP2‐Q83X), was used. Compared with RTT‐neurons_(Q83X)_ (non‐edited), edited RTT‐neurons with MeCP2‐gRNA (RTT‐neurons_(Q83Q)_) showed a significant increase in the expression of MeCP2 protein. In particular, the expression level of MeCP2 in edited RTT‐neurons_(Q83Q)_ was improved (36.3% increase) via the MACS process, whereas no difference was observed in control‐gRNA treated RTT‐neurons_(Q83X)_. Furthermore, immunofluorescence images of the neurons showed that repaired MeCP2 was expressed and localized to the nuclei of edited RTT‐neurons_(Q83Q)_ (47.3%), similar to WT‐neurons. In contrast, no signal was detected in the nuclei of non‐edited RTT neurons_(Q83X)_ (Figure 4d). These results further support that the mutated MeCP2 gene was successfully replaced with the donor and expressed the full sequence of MeCP2 in RTT‐neurons that interact with organelles as normal.^[^ ^23^ ^]^

### 2.5 Confirming Functionality of MeCP2 in Repaired RTT‐Neurons

**Figure 5:**
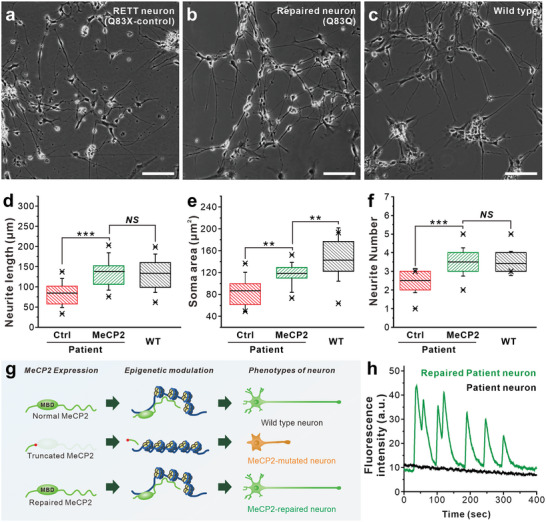
Improved phenotypes and functional recovery of repaired RTT‐neuron. a–c) Microscopic image of differentiated WT‐neurons (a), control RTT‐neurons (b), and repaired RTT‐neurons (c). Scale bar: 20 µm. d–f) Phenotypic analysis of edited RTT‐neurons in the length of neurites (d), soma size (e), and the number of neurites per cell (f) comparing to WT‐neurons and control RTT‐neurons. g) Schematic illustration of the phenotypic differences between WT, repaired and mutated RTT‐neurons. h) Calcium response of repaired RTT‐neurons monitored with fluorescence intensity recording and comparison with control RTT‐neurons.

To validate whether the observed changes in downstream gene expression resulted from “repaired” MeCP2 expression, we analyzed the resulting phenotype of the RTT‐neurons before and after genome editing. A fundamental morphological phenotype that is typically seen in RTT‐neurons_(Q83X)_ is the reduced complexity of the neuronal dendritic tree, which negatively impacts neural network development.^[^ ^24^ ^]^ To assay for neuronal functional recovery following editing of the mutated MeCP2 gene, the edited RTT‐neurons were compared with wild‐type neurons (**Figure** **5**). As expected, the WT‐neuron and “repaired” RTT‐neurons_(Q83Q)_ have a similar morphology (Figure 5a–c), whereas there was a significant difference in neurite length when compared with “mutated” RTT‐neurons_(Q83X)_ (Figure 5d). In particular, “repaired” RTT‐neurons_(Q83Q)_ showed up to 76.2% higher neurite outgrowth compared to “mutated” neurons_(Q83X)_. This result was confirmed to be directly related to the 2.7‐fold and 2.1‐fold improvement in the expression of BDNF and Reelin, genes that promote neurite outgrowth among the genes upregulated by MeCP2, respectively, in “repaired” RTT‐neurons (Figure S6a,b, Supporting Information).^[^ ^25^ ^]^ In addition, the size of the soma and the number of neurites in the “repaired” RTT‐neurons increased by up to 22.8% and 16.7%, respectively (Figure 5e,f). In particular, “repaired” RTT‐neurons showed improved dendritic growth of neurites, which was accompanied by a decrease in FXYD1 and DLX5 expression (Figures S6c,d, and S7, Supporting Information).^[^ ^26^ ^]^

Furthermore, we confirmed the functional recovery of RTT‐neurons that were edited using the MAGE‐CRISPR. In previous studies, neural cells from RTT patients were found to exhibit abnormal calcium transients compared to the WT group.^[^ ^9^, ^27^ ^]^ In particular, RTT‐neurons showed a significantly reduced calcium spike frequency.^[^ ^28^ ^]^ To this end, we monitored changes in intracellular calcium levels of treated and untreated cells as functionally active neurons should spontaneously fire action potentials that allow for the influx of cations, including calcium.^[^ ^29^ ^]^ To accomplish this, we used a commercially available calcium indicator dye, Fluo 4AM, to monitor changes in intracellular calcium concentrations via the visualization and quantification of fluorescence intensity. 21 days post‐differentiation, calcium imaging was performed. The calcium oscillation frequency of “repaired” RTT‐neurons expressing MeCP2 was dramatically improved compared to “mutant” RTT‐neurons, suggesting the restoration of neuronal activity dynamics (Figure 5h; Video S1, Supporting Information). When we directly compared the calcium influx and efflux of “repaired” and “mutated” RTT neurons through time‐lapse images, we could see no signal in the “mutated” RTT neurons (Figure S8, Supporting Information). These results suggest that edited RTT neurons exhibit functional activity.

## 3 Conclusion

In this work, a MAGE‐CRISPR was developed that significantly improves the genome editing efficiency of CRISPR‐Cas9. This MAGE‐CRISPR was composed of a MCNP with zinc‐doped iron oxide (ZnFe_2_O_4_) core and inert mesoporous silica shells coated with two layers of branched PEI to deliver multiple plasmids via electrostatic interaction. When using the CRISPR‐Cas9 system using DNA plasmid, there may be concerns about problems such as DNA insertion compared to using mRNA or ribonucleoprotein. However, it is worth noting that plasmids for non‐viral vectors have a significantly lower tendency for chromosomal integration compared to viral vectors, as reported in “USP35 <1047> Gene Therapy Products”.

Furthermore, to avoid any uncontrolled amplification of the exogenous donor template plasmid, we made sure to linearize the plasmids using restriction enzymes before their placement on the MAGE‐CRISPR. In addition to that, there have been reports stating that the utilization of linearized donor templates can enhance the efficiency of HDR.^[^ ^30^ ^]^ This could be achieved by generating sticky ends which facilitate the hybridization process between the homologous section of the donor DNA and the corresponding target. The risk of serialization using linearized plasmids and insertion into chromosomes is also recognized. However, the relative distance of the donor template from the potential linkage site minimizes the risk of chromosomal insertion or use of the wrong template. A notable HDR‐independent genome integration route involves directly linking linearized donor DNA ends to the truncated ends of genomic DNA. Recent studies have shown that inhibition of DNA‐dependent protein kinase (DNA‐PK) can reduce this process, and the strategy of co‐administration of M3814, a DNA‐PK inhibitor, may be considered in the future to alleviate potential problems.^[^ ^31^ ^]^

Several recent studies have reported attempts to use magnetofection to deliver plasmids in CRISPR applications.^[^ ^7^ ^]^ Compared to cationic polymer‐based approaches, this approach exhibits significantly improved efficiency in (specify the aspect of efficiency, e.g., delivery rate, cellular uptake, target gene editing). In addition, as another method utilizing magnetic properties, research has been attempted to enrich cells expressing specific surface markers using MACS,^[^ ^32^ ^]^ and the “magselectofection” method of applying magnetofection to specific cells separated by MACS has also been proposed.^[^ ^33^ ^]^ However, the findings presented in this paper represent a pioneering effort to achieve a significant proportion of modified cells. This was accomplished through the magnetic separation of cells that were introduced to a MAGE‐CRISPR via magnetofection. Remarkably, this approach resulted in over 40% of the cells successfully undergoing treatment with HDR. This achievement is noteworthy, as it surpasses the efficiency levels reported for HDR using other non‐viral vector‐based methods.^[^ ^19^, ^21^ ^]^

Non‐viral delivery methods often suffer from low editing efficiencies, particularly in stem cells, known for their low gene delivery efficiency. This challenge must be addressed before non‐viral CRISPR delivery can be effectively translated to the clinic for stem cell‐based therapies targeting genetic diseases.

To this end, our study demonstrates the effectiveness of MAGE‐CRISPR in enhancing genome editing efficiency, specifically in stem cells. By testing our method on stem cell models relevant to Rett Syndrome, we showcase its potential to overcome the low gene delivery efficiency that has hindered the progress of non‐viral CRISPR delivery in stem cell‐based therapies. The successful application of MAGE‐CRISPR in editing stem cells highlights its significance in advancing gene therapy for genetic disorders. Our results clearly suggest that MAGE‐CRISPR could be a powerful tool for developing stem cell‐based treatments for Rett Syndrome and other genetic diseases, offering a more efficient approach to genome editing in these challenging cell types. Our study demonstrates the potential of MAGE‐CRISPR to overcome the limitations of low editing efficiencies in stem cells. By focusing on the successful application of our method in stem cell models of Rett Syndrome, we highlight its significance in advancing stem cell‐based therapies for genetic diseases and its potential to revolutionize the field of gene therapy.

In conclusion, our research successfully shows the effectiveness of the multifunctional MAGE‐CRISPR in efficiently delivering CRISPR‐Cas9 to stem cells. After using the MAGE‐CRISPR to repair MeCP2, we observed a remarkable improvement in the phenotype of cells derived from patients. It is also worth noting that while we did not achieve 100% editing efficiency, cells with mutations still showed significant phenotypic advancements when cultured alongside corrected cells. This positive shift can be attributed to the exosomes secreted by the repaired cells, as mentioned in the reference.^[^ ^34^ ^]^ This presents a promising avenue for improving the functional aspects of patients with genomic disorders through ex vivo CRISPR treatment using the MAGE approach. In this method, the patient's cells are extracted first, then edited in a controlled laboratory setting, and finally, they are reintroduced back into the patient. Importantly, therapeutic benefits can be achieved without correcting every mutated cell. The potential of our developed nanobio‐combined CRISPR‐Cas9 technology can extend beyond addressing Rett syndrome, as it can be harnessed for a wide range of clinical applications, especially in stem cell therapy for genetic diseases.

## 4 Experimental Section

### Generation of MeCP2 Q83X‐iPSCs from Patients and Pre‐Differentiation into Neuron Progenitor Cells

The generated control (wild type, WT) and RTT (MeCP2, Q83X) iPSC clonal lines from skin fibroblasts were differentiated as previously described. Before the differentiation of the iPSC clonal lines into NPCs, cells were re‐plated at 30 000–40 000 cells per cm^2^ in N2/B27 medium without FGF2 and supplemented with 5 µm Y‐27632 (Stemgent, Cambridge, MA) and 1 µm retinoic acid (Tocris, Bristol, United Kingdom). Y‐27632 was withdrawn on day 3 after the plating of cells, and retinoic acid was withdrawn on day 7. Starting on day 3, the medium was supplemented with 200 µm ascorbic acid (Sigma‐Aldrich, St. Louis, MO), 1 µm dibutyryl‐cAMP (Sigma‐Aldrich, St. Louis, MO), 20 ng ml^−1^ BDNF (Life Technologies, Carlsbad, CA), and 20 ng ml^−1^ GDNF (Life Technologies, Carlsbad, CA) until day 10, after which basal NPC medium without FGF2 was used. The medium was partially changed every other day until day 21 or day 35 for further experiments. Both the RTT‐NPCs_(Q83X)_ and wild‐type NSCs were expanded for 3–5 passages in neural precursor medium (NPM) comprising a 1:1 ratio of DMEM/F12 and Neurobasal medium containing 20 ng ml^−1^ FGF2 and 0.5% B27 and N2. When the cells reached 90–100% confluency, they were passaged at a ratio of 1:2 on Matrigel using Accutase. The medium was changed every other day. The experimental protocol was approved by the Biosafety Committee of Rutgers (12‐325).

To induce neuronal differentiation, a fresh neuronal differentiation medium (NDM, NeuroBasal medium, 1% B27, 10 ng ml^−1^ BDNF) was added. The NDM was changed every three days. The gene expression was analyzed at Day 21 through qPCR and immunocytochemistry.

### Magnetic Core‐Mesoporous Silica Shell Nanoparticle Synthesis

Synthesis of Zinc‐doped Magnetic Nanoparticles, MNPs. The 25 nm with (Zn_0.4_Fe_0.6_)Fe_2_O_4_ magnetic cores were synthesized via modified established procedures from Cheon et al.^[^ ^10^ ^]^ Briefly, 1,2‐hexadecanediol (10 mmol), Fe(acac)_3_ (1.35 mmol), FeCl_2_ (0.7 mmol), and ZnCl_2_ (0.3 mmol) were added into a 100‐ml round bottom flask with 45.75 mmol of tri‐n‐octylamine, and 6 mmol of both oleic acid and oleylamine. Then, under a blanket of nitrogen, the reaction mixture was heated and maintained at 200 °C for 2 h. The mixture was then heated to 300 °C for 2 h. The formed nanoparticles were then allowed to cool down slowly to room temperature, after which they were collected by centrifugation at 10 000 rpm for 10 min and purified via repeated ethanol washings.

Synthesis of MNPs@Mesoporous Silica Core‐Shell Nanoparticles. To coat the magnetic nanoparticle cores with mesoporous silica, a modified procedure from the previous research was used.^[^ ^35^ ^]^ 5 mg of the alkly‐capped magnetic cores in chloroform was added to a 25‐ml solution of 0.1 m aqueous cetrimonium bromide (CTAB), followed by sonication via a probe‐type sonicator until the formation of a clear solution and evaporation of the chloroform. The CTAB capped‐magnetic core solution was then diluted to 50 ml, and the pH of this mixture was adjusted to pH 11 using 2 m NaOH. This mixture was heated to 70 °C under vigorous stirring, and 0.4 ml of TEOS in 2.4 ml ethyl acetate was added. After the addition of TEOS, the reaction was allowed to continue for 4 h. The MCNPs were collected and washed several times with ethanol. To remove the surfactant template, the nanoparticles were heated to 60 °C in an ammonium nitrate solution. The extracted MCNPs were again washed with ethanol. The product was confirmed using high‐resolution transmission electron microscopy (HR‐TEM), dynamic light scattering, and zeta potential measurement.

### Construction of pCas9‐MeCP2‐gRNA‐Puro and pDonor‐MeCP2‐EGFP Vectors

The study designed a gRNA sequence from the MeCP2 genome using the CRISPRgRNA software program (https://www.dna20.com). The designed specific MeCP2 targeting gRNA sequence (AUGAUGGAGCGCCGCUGUUU) was the best target sequence with significantly fewer off‐targets. An empty sequence of gRNA was used as a control. Two plasmid systems were constructed to edit the mutated MeCP2 genome: a combined Cas9 system with gRNA labeled pCas9‐MeCP2‐gRNA‐Puro and a donor sequence with the GFP gene named pDonor‐MeCP2‐EGFP. The plasmid backbones of gRNA and pCRISPR‐Cas9 were purchased from Addgene (Cambridge, Massachusetts) to construct pCas9‐MeCP2‐gRNA‐Puro. The pCas9 plasmid was updated using a bicistronic T2A gene to express both CRISPR‐Cas9 and the puromycin resistance gene cloned fused at the end of the Cas9 gene using the restriction enzyme PstI and PmeI: pCRISPR‐Cas9‐2A. The insert of U6 promoter‐MeCP2gRNA was then cloned into pCRISPR‐Cas9‐2A using MluI and SpeI restriction enzymes as the final plasmid construction of the pCas9‐MeCP2‐gRNA‐Puro. The pDNA4/TO plasmid was used as the backbone of the pDonorGFP plasmid. The designed donor sequences were 700‐base‐pair up and down‐stream from the double‐strand cleavage site by Cas9 and guide RNA complex. The first step was to clone the insert of CAG‐GFP into the backbone pDNA4/TO plasmid using the restriction enzymes, SpeI and HindIII. Next was to clone 700‐base‐pair downstream side of the donor sequences from the total of 1400 base pairs using MfeI and SpeI for the vector to open the plasmid and insert EcoRI and SpeI, which EcoRI is a compatible enzyme of MfeI. The next step was to clone the upstream part of the donor MeCP2 gene sequence. Ten base pairs were modified to differ from the gRNA target sequence at the mutated site. This was done using a compatible gene sequence for the wild‐type sequence to protect the donor plasmid from the MeCP2gRNA targeting: named pDonor‐MeCP2‐EGFP (pDonor). pCas9‐Cont‐gRNA‐Puro was an empty control plasmid, which was created without a gRNA sequence. To improve the HDR efficiency and prevent genomic DNA contamination during the next‐generation sequencing process, pDonor was linearized with BlpI by cutting 85 base pairs upstream from the Cas9 cut site. All plasmids were verified by sequencing before the genes were delivered to RTT‐NPCs_(__Q83X)_.

### Preparation of the MAGE‐CRISPR

To prepare the aforementioned MCNPs as a transfer vehicle for the MAGE‐CRISPR, the negatively charged MCNPs were coated with a TRITC‐labeled branched cationic polymer, polyethyleneimine (PEI, Mn. 10 kDa). TRITC (Thermo Scientific, MA, USA) and PEI were conjugated using an EDC/NHS reaction at a 1:1 molar ratio. The TRITC‐labeled PEI was added to a 1 mg ml^−1^ MCNP solution, resulting in a final concentration of 10 mg ml^−1^. Simultaneously, a NaCl solution was added to achieve a final concentration of 1 mm. This PEI coating condition was chosen based on previous reports.^[^ ^11b^ ^]^ The solution was stirred for at least 30 min and then purified by centrifugation at 10 000 rpm for 10 min. To remove excess PEI, the washing step was performed twice. For plasmid complexation, the plasmid solution and the TRITC‐PEI‐coated MCNPs were mixed in a mass ratio ranging from 1:1 to 1:30 (plasmid: MCNP) and incubated for 30 min at room temperature. Finally, the outer PEI layer was applied under the same conditions used for the TRITC‐labeled PEI coating.

### Plasmid Delivery Via MAGE‐CRISPR

The sequence‐verified, endotoxin‐free plasmids, pCas9‐MeCP2‐gRNA‐Puro and, pCas9‐Cont‐gRNA‐Puro with, and pDonor‐MeCP2‐EGFP were transfected into RTT‐NPCs_(Q83X)_ plated on a Matrigel‐coated (Life Technologies, Carlsbad, CA) dish via MAGE‐CRISPR. Twenty‐four hours before the magnetofection of MAGE, 3×10^5^ RTT‐NPCs_(Q83X)_ were seeded into each well of a 6‐well culture dish. After that, the MAGE was mixed with Opti MEM (Life Technologies) and added to each well to attain the desired final concentration of MAGE. Subsequently, the cell culture plates were placed on a Nd‐Fe‐B magnetic plate (OZ Biosciences, France) for 10–15 min (as optimized from previous reports).^[^ ^11^, ^36^ ^]^ The culture plates were placed back into the incubator for four hours and afterward, the cells were washed with DPBS, and the Opti MEM was replaced with a fresh growth medium. The growth mediums for the cell lines (obtained from ATCC) used in the study are as follows: Neuroprecursor Medium (DMEM/F12: Neurobasal Medium = 1:1), 0.5% N‐2 supplement, 0.5% B‐27, and 20 ng ml^−1^ bFGF.

To compare the delivery efficiency of the MAGE‐CRISPR with the traditional method, gene delivery was performed using Lipofectamine 3000 (Invitrogen, CA, USA) and plasmid DNA at a ratio of 3:1 according to the manufacturer's instructions. The cells were EGFP^+^ of ≈20–30% at 36 h post‐gene delivery (data not shown). Gene delivery was efficient in RTT‐NPCs_(Q83X)_, as RTT‐NPCs_(Q83X)_ proliferated well. The cells were then treated with 0.1 µg ml^−1^ puromycin for the first two times while changing the medium every other day, and then with 0.4 µg ml^−1^ until the non‐transfected cells were completely killed off in about two weeks after gene delivery. Neurite length and soma size were analyzed semi‐automatically using ImageJ software 2 to 3 days after the induction of the gene expression.

### Immunofluorescence imaging

All fluorescence images were obtained using a Nikon Eclipse Ti‐E inverted fluorescence microscope. To monitor the MAGE uptake into the RTT‐NPCs_(Q83X)_ and expression of delivered plasmids in RTT‐NPCs_(Q83X)_ in situ, cells were incubated on the stage‐top incubator of fluorescence microscope with 5% CO_2_ pre‐mixed gas and 37°C.

### Quantification of mRNA Expression

The total RNA was extracted from tissue culture using 1 mL of TRIzol Reagent (Life Technologies, CA, USA) following the manufacturer's protocol. The total RNA concentration was measured by NanoDrop LITE (Thermo Scientific, MA, USA) before performing reverse transcription using Superscript III First‐Strand Synthesis System (Invitrogen, CA, USA). Briefly, the amount of 1 microgram of purified total RNA was primed with oligo(dT) and converted to complementary DNA (cDNA) in a 20 µL reaction, according to the manufacturer's protocol. Then, the cDNA was diluted with 1:50 factor and subjected to quantitative PCR (qPCR) analysis with the gene‐specific primers, listed in Table S1 (Supporting Information). The qPCR reactions were performed on a StepOnePlus Real‐Time PCR System (Applied Biosystems, CA, USA) using *Power* SYBR Green PCR Master Mix (Applied Biosystems, CA, USA). The fold change in gene expression was calculated based on the resulting C_t_ values of the gene of interest relative to those of endogenous control (GAPDH). Standard cycling conditions were used for all reactions with a melting temperature of 60 °C. All primers were obtained from the PrimerBank database (Table S1, Supporting Information).^[^ ^37^ ^]^

### Next‐Generation Sequencing Analysis of MAGE‐Edited Cells

Prior to DNA extraction, the cells were trypsinized and re‐suspended in Dulbecco's Phosphate‐Buffered Saline (DPBS; Life Technologies, CA, USA), followed by the addition of Proteinase K (QIAgen Inc., MD, USA). Subsequently, the cell suspension was subjected to DNA isolation following the manufacturer's instructions of the QIAamp DNA Mini Kit (QIAgen Inc., MD, USA). The extracted DNA was used as a template to amplify the genomic region of HDR target sequence by Polymerase Chain Reaction (PCR) using Platinum SuperFi PCR Master Mix (Invitrogen, CA, USA) with Forward Primer: 5′‐ATCAGCCCACCACTCTGCT‐3′ and Reverse Primer: 5′‐CCCTGCCCTGTAGAGATAGGA‐3′ (Integrated DNA Technologies, Inc., IA, USA). The PCR reaction was purified using DNA Clean & Concentrator‐25 PCR Purification Kit (Zymo Research, CA, USA) and measured concentration using NanoDrop LITE (Thermo Scientific, MA. USA). The purity of DNA amplicon was assessed before next‐generation sequencing by 2% agarose gel electrophoresis in TAE buffer, stained with GelRed (Biotium, CA, USA). The gel was analyzed using iBright FL1000 (Invitrogen, MA, USA).

DNA library preparations and amplicon sequencing were conducted at GENEWIZ, Inc. (South Plainfield, NJ, USA). DNA amplicon was indexed and enriched by limited‐cycle PCR. The DNA library was validated using TapeStation (Agilent Technologies, CA, USA) and was quantified using Qubit 2.0 Fluorometer and real‐time PCR (Applied Biosystems, CA, USA). The pooled DNA libraries were loaded on the Illumina instrument according to the manufacturer's instructions. The samples were sequenced using a 2×250 paired‐end (PE) configuration. Image analysis and base calling were conducted by the Illumina Control Software (HCS) on the Illumina instrument.

Bioinformatics analysis was carried out on the FASTQ file using the CRISPResso2 tool^[^ ^38^ ^]^ with the following parameters: No filters for all quality filtering and trimming.

### Off‐Target Analysis Using T7 Endonuclease I Assay

The pools of CRISPR targets were identified using CCTop – CRISPR/Cas9 target online predictor tool.^[^ ^39^ ^]^ The off‐target gene candidates with total mismatches upto four were selected for subsequent off‐target analysis. For the T7E1 assay, the off‐target regions were amplified from isolated genomic DNA using AmpliTaq Gold 360 Master Mix with the PCR primers listed in Table S2 (Supporting Information). The cleavage was detected by GeneArt Genomic Cleavage Detection (GCD) Kit (Invitrogen, CA, USA), according to the manufacturer's protocol. The percentage of indels was calculated from the band intensities quantified by Image J software.

### Quantitative PCR Analysis

To determine the intracellular amount of plasmid DNA, 30 nanograms of isolated DNA was amplified on a StepOnePlus Real‐Time PCR System (Applied Biosystems, CA) using Power SYBR Green PCR Master Mix (Applied Biosystems, CA, USA) with primers designed to target a specific sequence on the Donor plasmid DNA (Forward 5′‐ AGCAGAAGAACGGCATCAAGGTGAACT‐3′; Reverse 5′‐ AGGTAGTGGTTGTCGGGCAGCA‐3′, Amplicon size = 136 bp). The amount of plasmid was quantitated based on the standard curve prepared by 1:10 serial dilution of the standard solutions of Donor plasmid DNA. All measurements were performed in triplicate.

### Western Blot Analysis

MAGE‐treated and non‐treated cells were trypsinized and re‐suspended in Dulbecco's Phosphate‐Buffered Saline (DPBS; Life Technologies, CA, USA). Collected cell pellets were lysed with sodium dodecyl sulfate (SDS) lysis buffer. Protein samples were separated by SDS‐polyacrylamide gel electrophoresis and transferred to polyvinylidene fluoride (PVDF) membranes (Millipore, Billerica, MA, USA). Protein‐transferred membranes were processed according to the ECL Western Blotting Protocol. Anti‐human MeCP2 (sc‐137070, Santa Cruz Biotechnology, TX, USA, 1:1000 dilution) antibody and β‐actin (#4967, Cell Signaling Technology, Boston, MA, USA, 1:1000 dilution) antibody were used as primary antibodies. All Western blot analysis was performed using the NIH ImageJ software.

### Calcium Imaging

Fluorescent calcium indicator dye Fluo4 AM (Life Technologies) was used for calcium imaging experiments. The dye was dissolved in DMSO and added to the cell culture to achieve a final concentration of 2 µm. Cells were incubated for 20 min with Fluo4 AM dye. Free dye was washed out with pre‐warmed HBSS (Life Technologies). Cells then were incubated for 30 min in DMEM media (Life Technologies) for de‐esterification of the Fluo4 AM dye. The media was replaced with pre‐warmed HBSS during the imaging session. Images were acquired using a Zyla sCMOS camera (Andor) mounted on an optical microscope (Nikon Eclipse Ti‐E) using a 20x, 0.75 NA objective. Images were taken at 0.5 s intervals for 1 min. Calcium imaging movies were displayed at 20 Hz. Images for calcium imaging were processed, and intensity was quantified using the NIH ImageJ software.

### Analysis of Neurite Length, Soma Size, and Neurites Number

Neurite length, soma size, and the number of neurites per cell were measured from 5–7 randomly taken images of total 240–400 cells. Cell adhesion survival was analyzed after selection by puromycin for two weeks between the groups of pCas9‐MeCP2‐gRNA‐Puro+pDonor‐MeCP2‐EGFP and pCas9‐Cont‐gRNA‐Puro+pDonor‐MeCP2‐EGFP as a control. The images were obtained randomly from each experimental group in the corresponding areas on the dish, and the numbers of attached EGFP+ cells were quantified via the ImageJ software program.

### Statistical Analysis

All the data are presented as the group mean values with the standard error of the mean (SEM). The statistical significance of differences in neurite length, soma size, and the number of neurites for each group was estimated using t‐test analysis. *p*‐values < 0.05 were considered statistically significant.

### Ethics

The experimental protocol was approved by the Biosafety Committee of Rutgers.

## Conflict of Interest

The authors declare no conflict of interest.

## Supporting information

Supporting Information

Supporting Information

## Data Availability

The data that support the findings of this study are available from the corresponding author upon reasonable request.
