# Supplementary material for: Magnetic Nanoparticle‐Assisted Non‐Viral CRISPR‐Cas9 for Enhanced Genome Editing to Treat Rett Syndrome
Source: Adv Sci (Weinh). 2024 Apr 22;11(24):2306432. doi: 10.1002/advs.202306432 (PMC11200027; doi:10.1002/advs.202306432)
Supplement: Supplementary file 1 — Supporting Information [file ADVS-11-2306432-s001.pdf]

ADVANCED SCIENCE
Open Access

Supporting Information

for *Adv. Sci.*, DOI 10.1002/advs.202306432

Magnetic Nanoparticle-Assisted Non-Viral CRISPR-Cas9 for Enhanced Genome Editing to Treat Rett Syndrome

Hyeon-Yeol Cho, Myungsik Yoo, Thanapat Pongkulapa, Hudifah Rabie, Alysson R. Muotri, Perry T. Yin, Jeong-Woo Choi and Ki-Bum Lee**

SUPPLEMENTARY INFORMATION

Magnetic Nanoparticle-Assisted Non-Viral CRISPR-Cas9 for Enhanced Genome Editing to Treat Rett Syndrome

Hyeon-Yeol Cho†, Myungsik Yoo†, Thanapat Pongkulapa†, Hudifah Rabie, Alysson R. Muotri, Perry T. Yin, Jeong-Woo Choi*, and Ki-Bum Lee*

SUPPLEMENTARY FIGURES AND TABLES		Page
Table S1	Table of the primers used for quantitative PCR.	S-2
Table S2	Table of the primers used for off-target analysis.	S-2
Supplementary Figure 1:	Schematic illustration of the sequence at the MeCP2 Q83X nonsense mutated site to correct the mutation using a CRISPR-Cas9 system.	S-3
Supplementary Figure 2:	Design of plasmids and guide RNAs.	S-4
Supplementary Figure 3:	Plasmid loading capacity and cytotoxicity of MAGE	S-5
Supplementary Figure 4:	Expression profiles of Delivered plasmid with different methods	S-6
Supplementary Figure 5:	Quantification of MAGE and plasmid in iPSC-NPCs after the magnetofection (MF) and cell sorting by MACS.	S-7
Supplementary Figure 6:	RT-PCR analysis of downstream genes of MeCP2.	S-8
Supplementary Figure 7:	The dendritic growth of neurites after the MeCP2 gene repairing.	S-9
Supplementary Figure 8:	Calcium response of a repaired RTT-neuron monitored with time-lapse imaging.	S-10

Targets	Forward Primer (5’-3’)	Reverse Primer (5’-3’)
GAPDH	CCGCATCTTCTTTTGCGTCG	GCCCAATACGACCAAATCCGT
BDNF	GGCTTGACATCATTGGCTGAC	CATTGGGCCGAACTTTCTGGT
Reelin	ACATCTACAAGTGTTCAGGCATC	TGGTTACCAAACTGGTGGTCA
FXYD1	CACGACCCGTTCACTTACGAC	ATCTTCTGCTCAGCACGATGA
DLX5	CTACAACCGCGTCCCAAG	GGTTTGCCATTCACCATTCT

Table S1. Table of the primers used for quantitative PCR. All primers were obtained from the PrimerBank database[1-3].

Off-target	Sequence	Forward Primer (5’-3’)	Reverse Primer (5’-3’)	Size (bp)
GUCY1A2	ATGATGAAGCACCACTATTT	ACCAGCTAGTATGACCTGAA	GCTATCTCATCAACTGTCGTC	472
NT5E	CTGCTGGTGCGCCGCTCTTT	GTAGACTTCGTGCGTTCTC	GAAGAGTGGAGAGGTTGTTC	410
TRAPPC9	ATGGAGGAGCCCAGCTATTT	GCCATGAATGTAGGGATCTTG	ATGGCTTTGGATGCTTGG	467

Table S2. Table of the primers used for off-target analysis. All primer sequences were designed using GenScript Online PCR Primers Designs Tool. The underlined letters indicated the mismatch sites.

REFERENCES FOR THE SUPPORTING INFORMATION:

1. Wang, X. A PCR primer bank for quantitative gene expression analysis. *Nucleic Acids Research* **31**, 154e-154 (2003).
2. Spandidos, A. et al. A comprehensive collection of experimentally validated primers for Polymerase Chain Reaction quantitation of murine transcript abundance. *BMC Genomics* **9**, 633 (2008).
3. Spandidos, A., Wang, X., Wang, H. & Seed, B. PrimerBank: a resource of human and mouse PCR primer pairs for gene expression detection and quantification. *Nucleic Acids Res* **38**, D792-799 (2010).

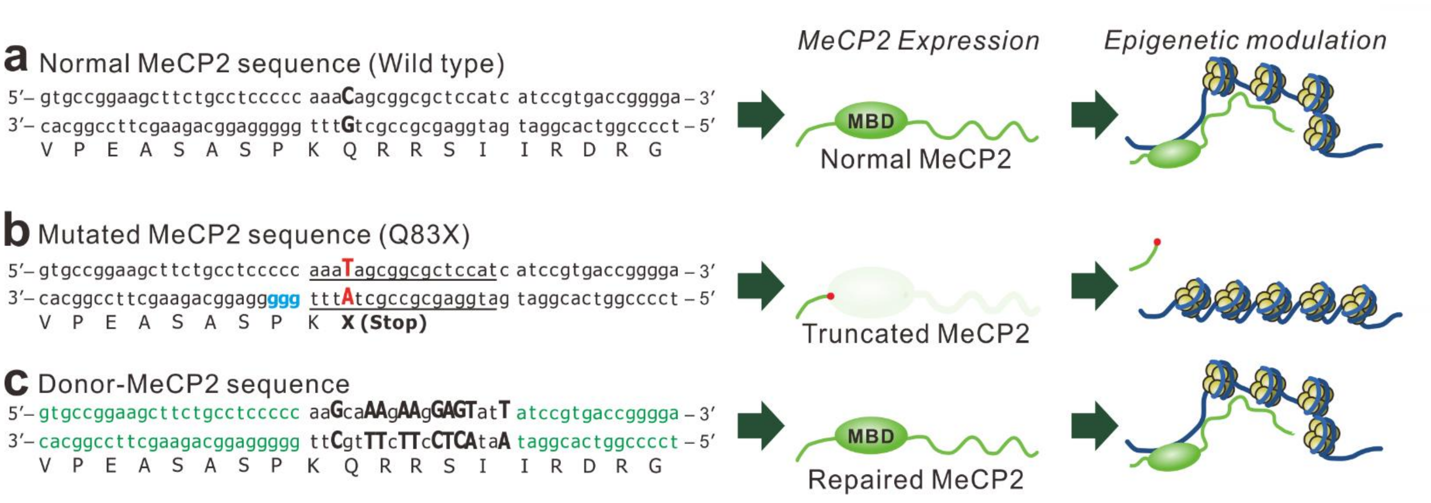

Figure S1. Schematic illustration of the sequence at the MeCP2 Q83X nonsense mutated site to correct the mutation using a CRISPR-Cas9 system. (**a**) The 83rd amino acid codon in the wild type MeCP2 gene sequence is CAG, which codes for glutamine (Q) as shown in bold capital letters below the gene sequence and the deduced amino acid sequence. (**b**) The MeCP2 mutated Rett syndrome gene sequence at the 83rd amino acid is TAG, which stops protein expression as shown with a bold capital letter X in the sequence. (**c**) To protect the donor plasmid and corrected gene from the continuous expression of Cas9 and MeCP2-gRNA, the gene sequence of the donor has been changed to a compatible gene sequence for the same amino acid as shown in capital letters in the sequence.

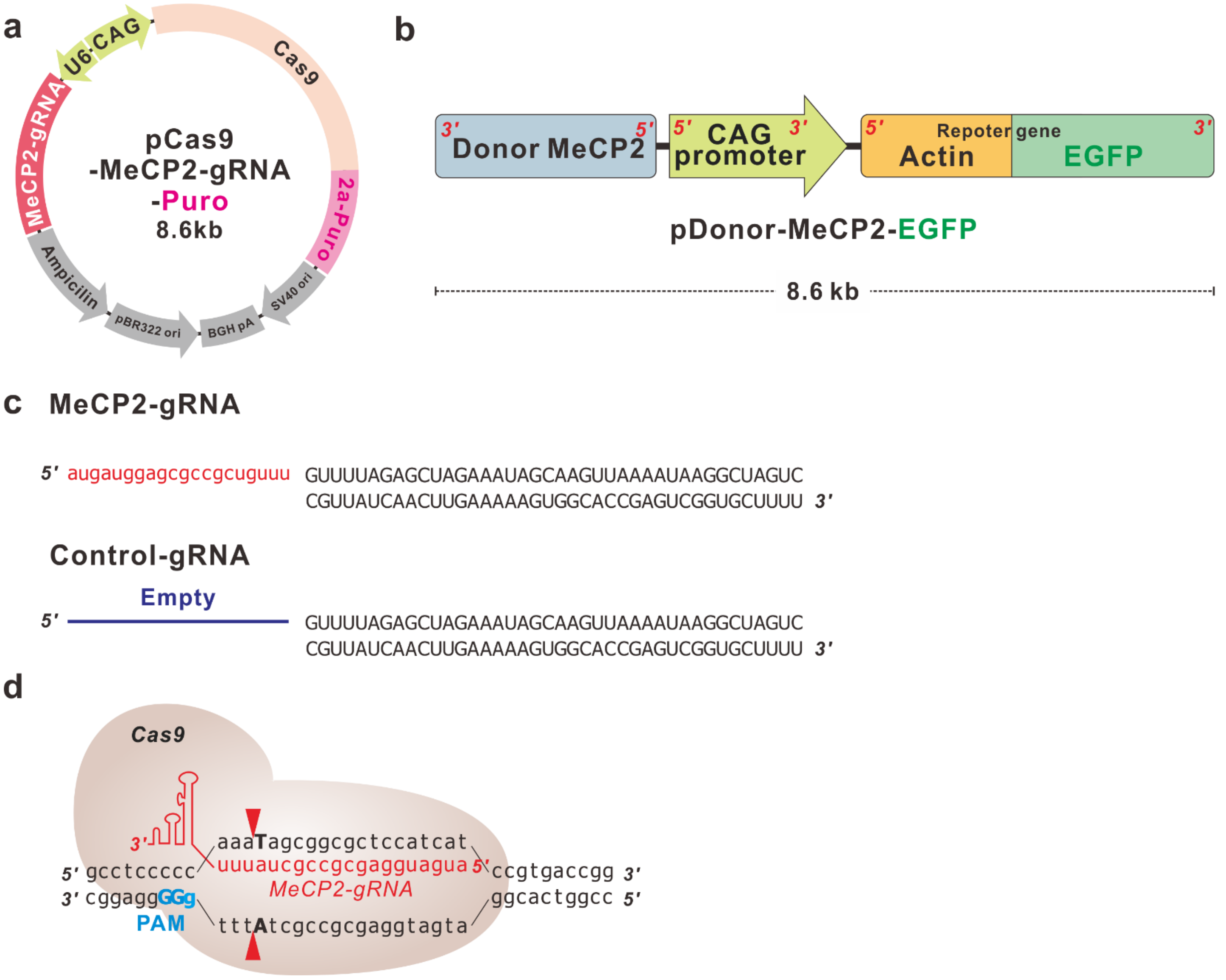

Figure S2. Design of plasmids and guide RNAs. (**a**) pCas9-gRNA-Puro, a combined single plasmid vector system based on the plasmid pcDNA^{TM}4/TO, whose expression is driven by a U6 promoter, has the target guide RNA sequence for MeCP2. A chicken beta-actin (CAG) promoter drives the expression of Cas9 fused with the puromycin resistance gene, which allows for the selection of transfected eukaryotic cells using T2A for bicistronic gene expression. (**b**) Linearized pDonor-MeCP2-EGFP is the plasmid vector system that contains the donor sequence for the MeCP2 and a CAG promoter that drives the expression of the enhanced green fluorescence protein (EGFP) gene to identify transfected cells. (**c**) MeCP2-guide RNA sequence is designed to target the mutated sequence of MeCP2 specifically for the Q83X patient. The empty guide RNA sequence is designed as a control. (**d**) MeCP2-gRNA bound at the MeCP2 nonsense mutated site recruits Cas9. After Cas9 binds to the MeCP2-gRNA, Cas9 cleaves the double-stranded MeCP2 gene 3-base-pair downstream at the Protospacer Adjacent Motif (PAM) sequence NGG (blue labeled sequence). The mutated MeCP2 gene target and Cas9 recruiting gRNA sequences are shown in small and capital letters, respectively.

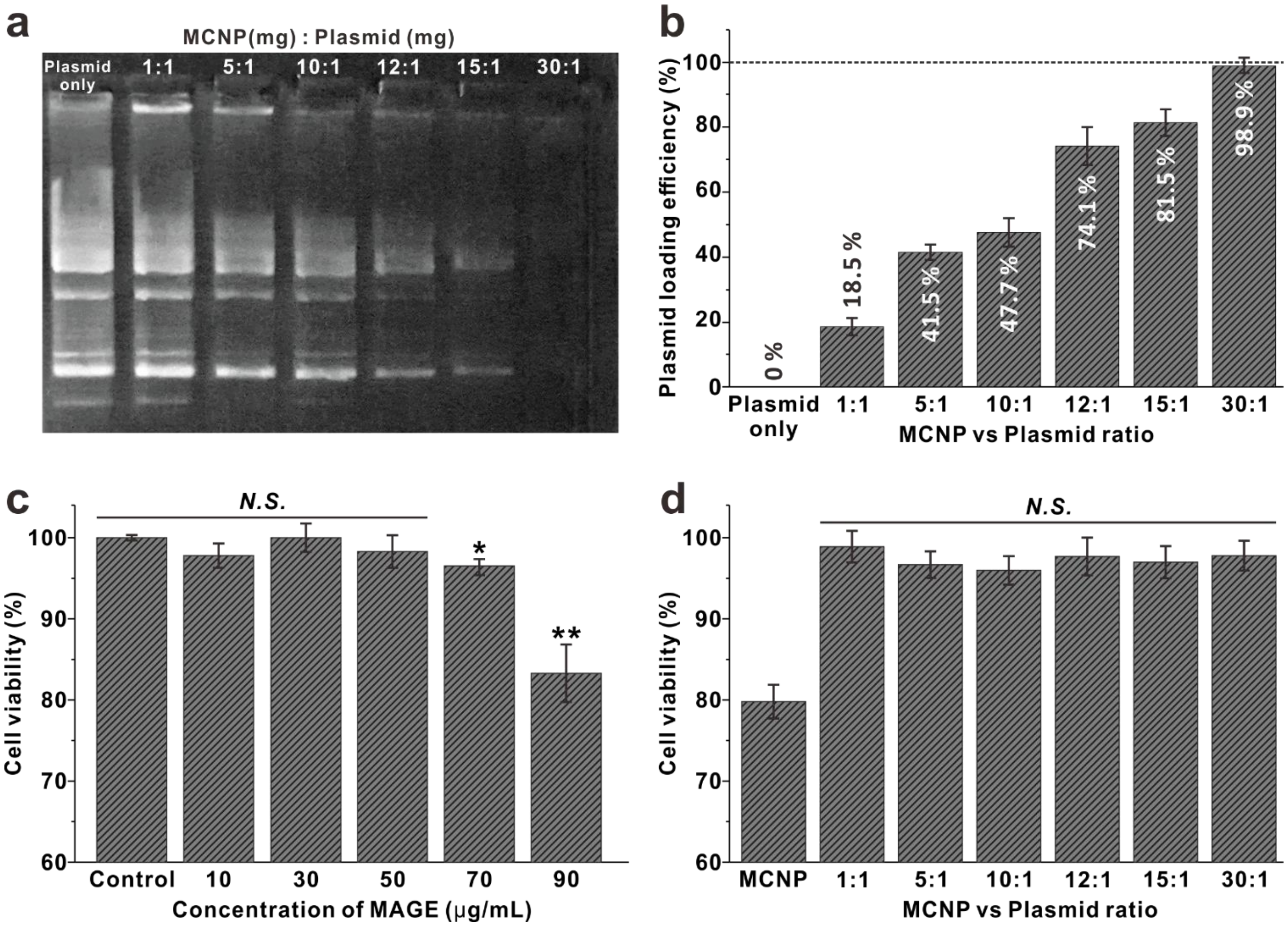

Figure S3. Plasmid loading capacity and cytotoxicity of MAGE. (**a**) Gel electrophoresis image of the remaining amounts of plasmids after the loading on the magnetic core-shell nanoparticle (MCNP). (**b**) The plasmid loading efficiency was calculated from (**a**). (**c**) Cytotoxicity of MAGE with the fixed ratio between MCNP and plasmids. (**d**) Cytotoxicity of MAGE with the different ratios between MCNP and plasmids related to the net charge of MAGE.

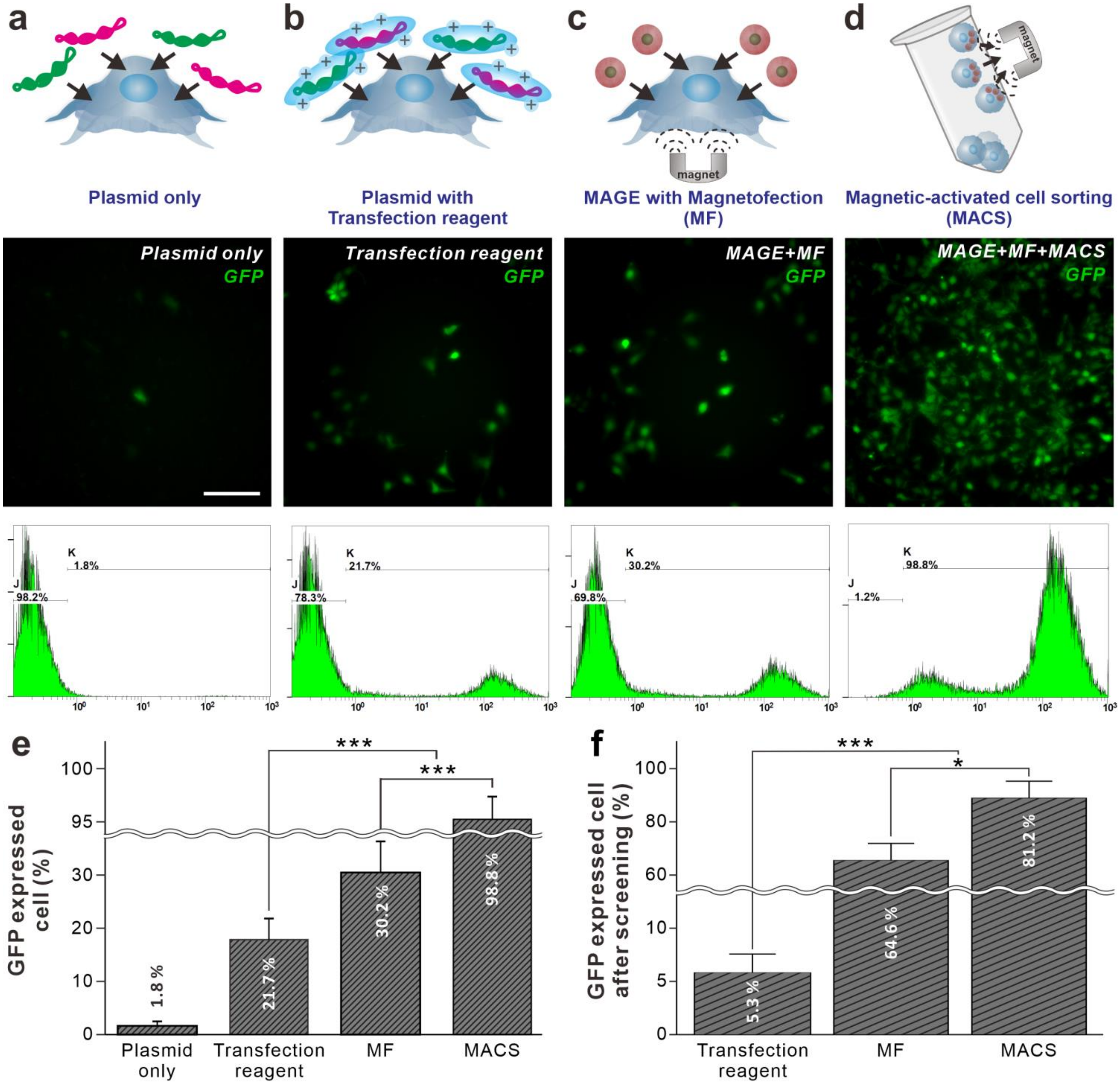

Figure S4. Expression profiles of Delivered plasmid with different methods. (**a-e**) Fluorescence images of the GFP expressed iPSC-NPCs after the plasmid delivery; (**a**) plasmid only, (**b**) transfection reagent, (**c**) MAGE with magnetofection, and (**d**) MAGE with magnetofection (MF) and magnetic-activated cell sorting (MACS). (**e**) Statistical analysis of GFP expression with different plasmid delivery methods. **f,** Quantification of EGFP expressed cells population 24 hours after puromycin screening (t-test, $^*P < 0.05$, error bars: s.e.m.).

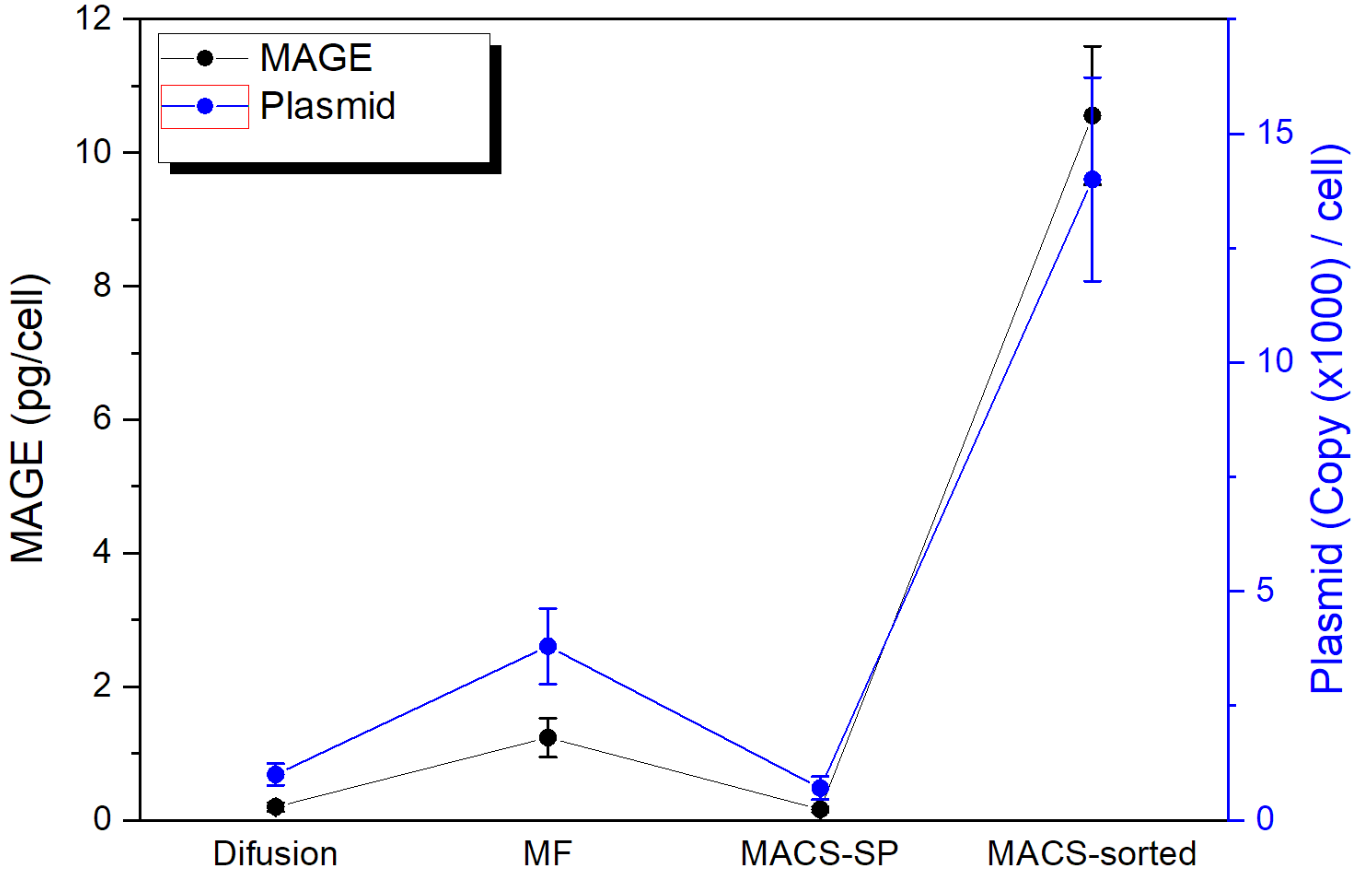

Figure S5. Quantification of MAGE and plasmid in iPSC-NPCs after the magnetofection (MF) and cell sorting by MACS. The amount of MAGE in an iPSC-NPCs was measured by ICP-OES and analyzed with iron ion's concentration per each cell. The copy number of plasmid per cell was calculated with a standard curve between Ct value and the copy number of plasmid from qPCR.

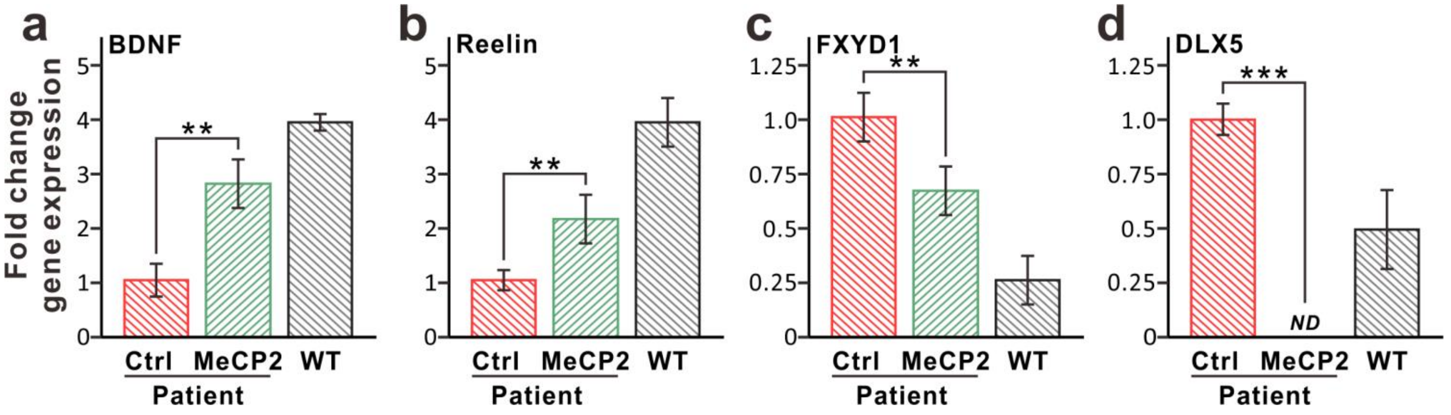

Figure S6. RT-PCR analysis of downstream genes of MeCP2; up-regulation: BDNF (a), Reelin (b), and down-regulation: FXYD1 (c), DLX5 (d). (t-test, **P < 0.01, ***P<0.001, error bars: s.e.m.). BDNF, brain-derived neurotrophic factor; FXYD1, FXYD domain containing ion transport regulator 1; DLX5, distal-less homeobox 5.

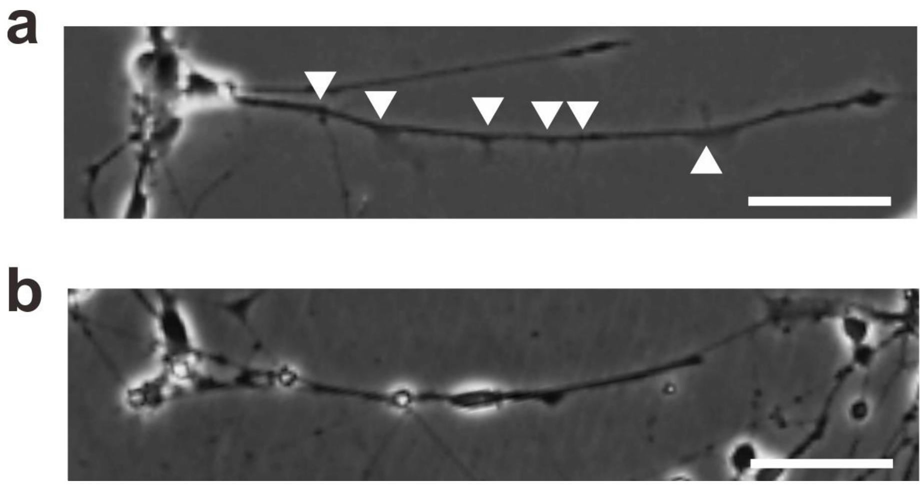

Figure S7. The dendritic growth of neurites after the MeCP2 gene repair. MeCP2 repaired neurons with (**a**) MeCP2-gRNA and (**b**) control-gRNA. White triangle: outgrown dendrite.

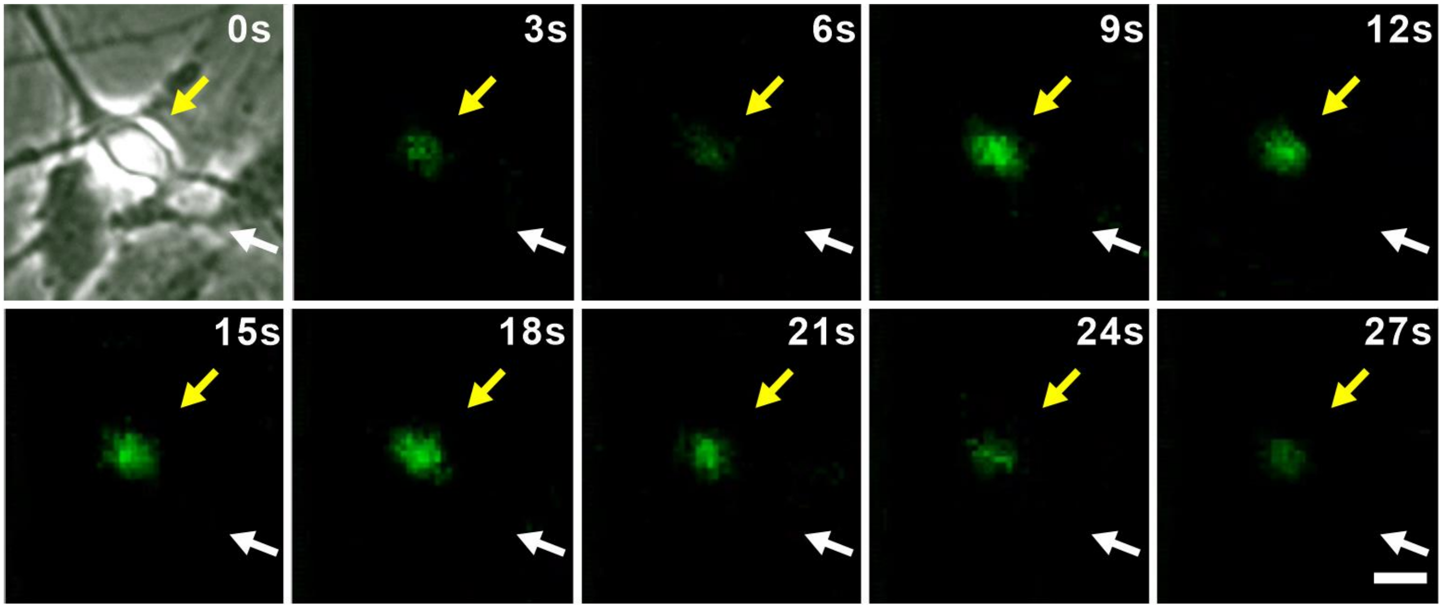

Figure S8. Calcium response of a repaired RTT-neuron monitored with time-lapse imaging. The 'repaired' RTT-neuron$_{(Q83Q)}$ indicated in the yellow arrow and 'mutated' RTT-neuron$_{(Q83X)}$ indicated in the white arrow. Scale bar: 10 μm.
